# Primary Synovial Sarcomas of the Mediastinum: A Systematic Review and Pooled Analysis of the Published Literature

**DOI:** 10.1155/2014/412527

**Published:** 2014-01-20

**Authors:** Samer Salah, Ahmed Salem

**Affiliations:** ^1^Department of Medical Oncology, King Hussein Cancer Center, Queen Rania Street, Al-Jubeiha, Amman 11941, Jordan; ^2^Department of Radiation Oncology, King Hussein Cancer Center, Queen Rania Street, Al-Jubeiha, Amman 11941, Jordan

## Abstract

*Background*. The aim of this systematic review is to attempt to provide a descriptive analysis for cases of synovial sarcoma (SS) arising in the mediastinum and to analyze prognostic factors. *Methods*. We performed PubMed database search in July 2013. Twenty-two studies, which included 40 patients, form the basis of this review. Demographic and disease-related factors were analyzed for possible influence on survival. Findings were compared with extremity SS studies reported in literature. *Results*. Sixteen cases (40%) presented with locally advanced unresectable disease, 2 (5%) with metastatic disease, and 22 (55%) with localized resectable disease. Median tumor size was 11 cm (range: 5–20 cm). Thirty patients were assessable for survival and had a 5-year OS of 36%. Completeness of resection was the only factor associated with significant improvement in OS (5-year survival of 63% and 0% in favor of complete resection, *P* = 0.003). *Conclusion*. Mediastinal SS is associated with poor prognosis as more cases are diagnosed at an advanced stage and with larger tumor size compared to extremity SS. Complete surgical resection is the only identified factor associated with better prognosis and may result in survival outcomes that are comparable with those for localized SS of the extremity.

## 1. Introduction

The mediastinum is a host for a variety of primary and secondary malignancies. Metastatic carcinomas, lymphomas, and thymomas are the most commonly encountered tumors. Synovial sarcoma (SS), though very rarely encountered, could also arise in this location [1–8].

Around 80% of synovial sarcomas arise in the extremities [9, 10]. In that context, prognostic factors for survival and recurrence are well characterized. Tumor size of ≥5 cm [11–17] and completeness of resection [16, 18, 19] are the most consistent prognostic factors among previous reports. Other factors, though less consistently identified, include bone or neurovascular invasion [14, 20, 21], high histologic grade [20, 21], and the histologic subtype [15, 19]. It is not clear if these prognostic factors retain their significance in cases of mediastinal SS. Furthermore, the distinctive features that are unique to the mediastinal site of occurrence of those tumors as opposed to other sites, including the tendency to present with larger size, and their site within the mediastinum (anterior, middles, or posterior mediastinum) as well as the presence of pericardial invasion and effusions, make any application of the previously identified prognostic factors irrelevant.

In the current paper, we attempt to provide a descriptive analysis of mediastinal SS and the patterns of failure following therapy. Furthermore, we analyze factors that may be prognostic for survival.

## 2. Methods

A comprehensive PubMed search was conducted in July 2013. The following search terms were used: “synovial,” “sarcoma,” and "mediastinum.” No restrictions were applied to the date of publication; however, this search was limited to papers in English language. Reports describing mediastinal SS cases were considered. Furthermore, reference lists of included studies were hand-searched to identify relevant missing publications. Full text articles of eligible abstracts were reviewed. Data pertaining to the age at diagnosis, gender, extent of disease at initial presentation (localized, locally advanced, or metastatic), location of the tumor inside the mediastinum (posterior, middle, and anterior), maximum tumor dimension, histologic subtype (monophasic or biphasic), presence of pericardial effusion, therapeutic modalities (surgery, chemotherapy, and radiotherapy), and completeness of surgical resection were extracted using a predefined datasheet. In addition, the status of patient at last follow-up and sites of any recurrence or progression (if any) were accurately documented. Overall, twenty-two studies—which included 40 patients—form the basis of this review [1–8, 22–35].

### 2.1. Statistical Analysis

Overall and event-free survival (EFS) were defined as surviving and surviving without any recurrence or progression, respectively. The influence of possible prognostic factors on survival was assessed and compared through the Log-Rank test. Survival curves were plotted through the Kaplan-Meier method. A *P* value of <0.05 was considered statistically significant. All statistical analyses were performed using SPSS version 17 (SPSS Inc., Chicago, IL).

Statistical testing for heterogeneity was not performed since it requires studies with larger sample size and is therefore not applicable to the included studies in this review (a small number of patients in each individual paper).

## 3. Results

### 3.1. Descriptive Analysis

The characteristics of the 40 reviewed patients including demographic patient data, clinical and pathological disease variables, and the mode of presentation are outlined in Table 1.

Although a wide spectrum of presenting symptoms and signs were noted (chest symptoms, fever, fatigue and weight loss), more than three-quarters of patients presented with chest or shoulder pain with or without shortness of breath.

Follow-up data was available for 30 of the 40 included patients, and was included in the survival analysis.

### 3.2. Therapeutic Strategies

Complete resection was the most commonly applied therapeutic strategy (23 patients; 57.5%); in 8 of those patients the surgery was part of multimodality treatment including chemotherapy and/or external beam radiotherapy (EBRT). The other 17 patients (42.5%) were treated by incomplete resections or with chemotherapy and radiotherapy secondary to the advanced stage of disease at presentation as outlined in Table 2.

### 3.3. Patterns of Recurrence and Progression

Of the 30 patients with available follow-up data, 20 (67%) had progression of disease (PD) following the primary therapy: nine of 17 patients with localized disease and 11 of 13 of patients with advanced disease. The median time to progression was 18 months for patients with completely resected tumors and 6 months for patients who did not have complete resection.

The most common site of progression was locally within the mediastinum. Of 15 patients with available data on sites of progression, 6 (40%) had isolated progression in the primary mediastinal tumor site. Other sites of progression included isolated progression in the lungs (3 patients), multiple metastatic sites (3 patients), liver (1 patient), lungs and the primary mediastinal tumor site (1 patient), and dural metastases (1 patient).

### 3.4. Survival Outcome

**Figure 1:**
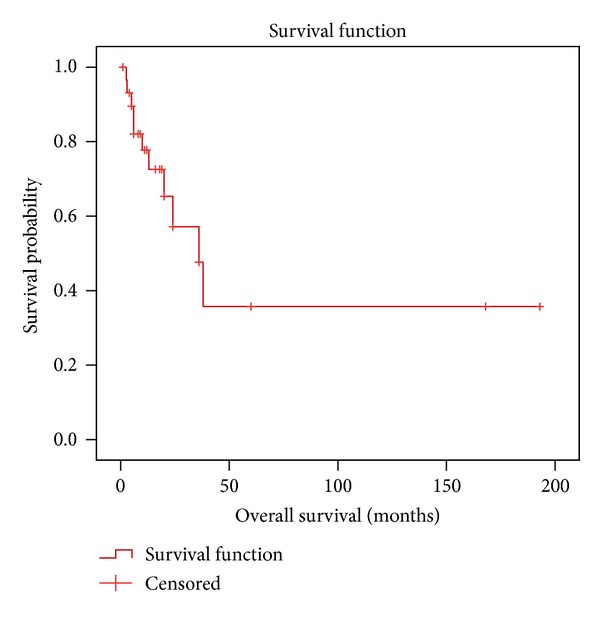
Kaplan-Meier overall survival estimation for the patients with mediastinal synovial sarcoma.

The mean follow-up time for the patients with reported outcome data was 26.8 months (range: 3–193 months). Eleven of those patients were deceased due to their disease, 9 were alive with evidence of disease, 8 were alive without evidence of disease, and two were alive with unknown disease status. The median EFS was 12 months with a 5-year EFS of 15%. The median overall survival was 36 months with a 5-year overall survival (OS) of 35.7% (Figure 1).

**Figure 2:**
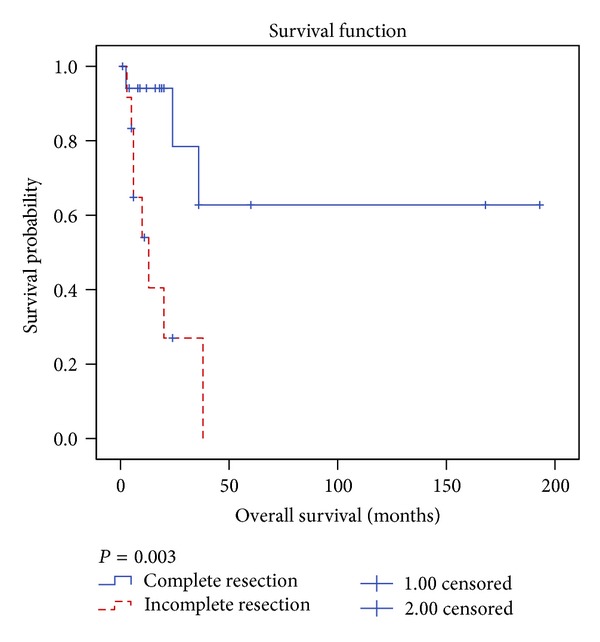
Kaplan-Meier overall survival estimation for patients with mediastinal synovial sarcoma according to completeness of surgical resection.

Using univariate analysis (Table 3), completeness of resection was identified as the only factor associated with statistically significant improvement in survival. The 5-year OS rate was 63% versus 0% in favor of complete resection, *P* = 0.003 (Figure 2). The other factors had no significant influence on survival.

## 4. Discussion

Less than 10–20% of SS arise in extra-extremity locations [9, 10]. Generally, Soft tissue sarcomas, including angiosarcoma, leiomyosarcoma, sarcomatoid mesothelioma, rhabdomyosarcoma, and SS, account for less than 0.01% of all malignant thoracic neoplasms [36]. According to data from a large population based study, around 17% of new cases of soft tissue sarcomas (including variety of histologic subtypes) arise in thoracic locations including the pleura, lungs, and the mediastinum with an approximate incidence of 6 per million populations [37]. Nevertheless, there are no data that accurately report the exact incidence of SS cases that arise primarily in the mediastinum.

Morphologically, synovial sarcomas are divided into monophasic and biphasic subtypes. The biphasic variant consists of proliferation of bland looking spindle-shaped cells, along with evidence of epithelial differentiation. Monophasic subtypes on the other hand can show either spindle cells only or occasionally epithelial component only [38]. A poorly differentiated variant of synovial sarcoma is also recognized [39]. Immunostains are valuable and supportive for the diagnosis of a suspected Case of SS. The expression of epithelial markers in the gland-like component and more importantly in the spindle cell component supports the diagnosis. EMA is the most commonly positive marker among all epithelial markers [39]. Pan-cytokeratin and cytokeratins 7 and 19 can also be positive in the epithelial-like component as well as the spindle cell component [40]. In addition, positivity for bcl-2 can be seen in some cases [41].

SS is characterized by unique t(X; 18)(p11.2; q11.2) translocation resulting in the fusion of the SYT gene on chromosome 18 to either of two closely related genes: SSX1 and SSX2 on chromosome X [42–44]. The availability of molecular testing (fluorescent in situ hybridization or polymerase chain reaction) of the t(X; 18) has improved diagnostic specificity for this disease as this translocation is found in over 90% of cases [45]. Testing for this translocation is important to confirm a suspected diagnosis of synovial sarcoma when it arises in a rare location such as the mediastinum.

The current review demonstrated important differences in the characteristics of these mediastinal tumors when compared to cohorts of SS of the extremities. Firstly, the median tumor size in the current analysis is 11 cm and none of the reported cases had a tumor size <5 cm. This size is well above the 5–7 cm median size reported in studies of SS where all or most patients have tumors in the extremities [17, 19, 46]. A likely explanation for this observed difference is that their mediastinal location allows them to grow to huge sizes before they become symptomatic. Secondly, a significant proportion of patients in the current analysis (43%) presented with advanced and unresectable disease as opposed to less than one-quarter of patients in the series that includes extremity sites as the sole or predominant site [12, 47]. Thirdly, the 5-year OS for the current cohort of patients (35.7%) is far below the 50–80% 5-year OS reported in studies that included patients with extremity SS [12, 47, 48]. The unsatisfactory survival results identified in the current analysis are clearly attributable to more advanced stage and larger tumor size at presentation; both are among the most consistently identified prognostic factors for SS. On the other hand, the 63% OS for patients with resectable disease in the current analysis approaches the reported OS of extremity SS who were treated with complete resection [46, 47, 49].

Unexpectedly, and as opposed to SS in other locations, we failed to reveal any prognostic significance based on tumor size in this analysis. This is probably because all patients in the current cohort had tumor size above the cutoff value (5 cm) which was most consistently identified as prognostic indicator for this tumor.

We emphasize the importance of complete resection of these tumors as completeness of resection was the only identified factor that is associated with improved survival. Other factors, including the location within the mediastinum, the presence of pericardial effusion, and the histologic subtype had no influence on survival. For that reason, patients should not be denied curative resection based on these factors as long as the tumor can be resected without residual disease.

Patients who present with unresectable nonmetastatic disease should also be managed with the aim of cure as those tumors are highly responsive to radiotherapy and chemotherapy and since obtaining adequate response may allow subsequent surgical resection [25]. It is worthy mentioning that SS is one of the sarcoma subtypes with heightened sensitivity to chemotherapy; response rates in the range of 30–55% have been consistently reported [50–53]. Specifically, high dose ifosfamide (12–18 g/m^2^/cycle) with or without doxorubicin appears to be associated with the highest reported objective response rate [53, 54] and might be an appropriate regimen for that particular indication.

The high proportion of patients who have subsequent local progression may suggest that adjuvant radiotherapy should be considered as part of a multimodality therapeutic approach for all patients. The role of chemotherapy remains elusive; however, some studies have suggested that adjuvant ifosfamide-based therapy improves survival of high risk SS of the extremities particularly those with tumor size of >5 cm [38]. We failed to show such survival advantage of chemotherapy in our study. It is likely that factors such as the small number of patients, the retrospective design, and the heterogeneity in chemotherapy regimens and patients' and tumors' characteristics are possible explanations.

An important limitation of our analysis is that it was performed for patients whose outcomes were reported from different studies; each one includes a limited number of patients, and as such, statistical tests of heterogeneity could not be performed to ensure the lack of heterogeneity among patients included from different studies. In addition, we cannot reliably exclude the possibility of publication biases with the tendency to report the patients who survived longer and to underreport those with suboptimal outcomes. Nevertheless, and given the rarity of mediastinal SS, no other formal attempt to analyze the influence of disease related factors on survival is realistically possible. Consequently, the current analysis can be utilized with caution as a guidance emphasizing the importance of complete resection of those tumors, the possible need for multimodality adjuvant therapy, and as an approximate estimation of recurrence and survival outcomes.

## 5. Conclusion

Mediastinal SS have poor prognosis as they tend to present with large tumors and with advanced stage. Completeness of resection is the only identified factor that influences survival and can result in outcomes similar to the outcomes following resection of SS arising primarily in the extremities.

## Figures and Tables

**Table 1:** Characteristics of the forty cases of mediastinal synovial sarcoma reported in the literature.

Variable	Result (range)
Median age	30.5 years (3–83 years)

Male : female ratio	2.9 : 1

Median size	11 cm (5–20 cm)

Status at presentation	
Localized resectable	22 (55%)
Locally advanced, unresectable	16 (40%)
Metastatic	2 (5%)

Location	
Anterior/middle mediastinum	28 (70%)
Posterior mediastinum	8 (20%)
Uncertain	4 (10%)

Histologic subtype	
Monophasic	18 (45%)
Biphasic	13 (33%)
Uncertain	9 (22%)

Surgical resection	
Complete resection	23 (58%)
No resection or partial resection	17 (42%)

Presenting symptoms and signs*	
Chest or shoulder pain	22/32 (69%)
Shortness of breath	20/32 (63 %)
Cough	5/32 (16%)
Pericardial effusion	7/32 (22%)
Pleural effusion	3/32 (9%)
Weakness and fatigue	4/32 (13%)
Fever	4/32 (13%)
Weight loss	3/32 (9%)
SVC obstruction	1/32 (3%)

SVC: superior vena cava.

*32 patients had clear data about clinical presentation.

**Table 2:** Therapeutic modalities for the 40 cases of mediastinal synovial sarcoma reported in the literature.

Treatment modality	Number of patients (%)
Complete resection only	15 (37.5%)
Complete resection + CTX + EBRT	4 (10%)
Complete resection + CTX	2 (5%)
Complete resection + EBRT	2 (5%)
Partial resection + EBRT	3 (7.5%)
Partial resection + EBRT + CTX	1 (3%)
CTX only	4 (10%)
EBRT only	4 (10%)
CTX and EBRT only	3 (7.5%)
BSC only	2 (5%)

CTX: chemotherapy; EBRT: external beam radiotherapy; BSC: best supportive care.

**Table 3:** Univariate analysis for the 30 cases of mediastinal synovial sarcoma with available follow-up data.

Variable	Number (%)	MS (months)	5-year OS	Log-Rank *P* value
Age				
≥20 years	24 (80%)	38	39.9%	0.32
<20 years	6 (20%)	24	31.3%	

Gender				
Male	24 (80%)	Unreached	56.2%	0.91
Female	6 (20%)	36 months	0%	

Pericardial effusion				
Yes	4 (13%)	Unreached	67%	0.83
No	26 (87%)	36 months	29%	

Histologic subtype				
Monophasic	10 (33%)	24 months	0%	0.11
Biphasic	10 (33%)	36 months	50.0%	
ND	10 (33%)			

Size				
<10 cm	8 (26%)	Unreached	75%	0.36
≥10 cm	11 (37%)	36 months	26%	
ND	11 (37%)			

Surgery				
Complete resection	17 (57%)	Unreached	63%	0.003
Incomplete	13 (43%)	13 months	0%	

Chemotherapy				
Yes	14 (47%)	36 months	46%	0.31
No	16 (53%)	24 months	24%	

Radiotherapy				
Yes	14 (47%)	36 months	21%	0.89
No	16 (53%)	unreached	53%	

Location in mediastinum				
Anterior/middle	22 (74%)	36 months	39%	0.93
Posterior	4 (13%)	6 months	50%	
ND	4 (13%)			

MS: median survival; OS: overall survival; ND: no data.
